# Breakdown of thalamocortical connectivity under sleep deprivation: implications for cognitive arousal and transient sleep states

**DOI:** 10.1093/sleepadvances/zpaf065

**Published:** 2025-10-01

**Authors:** David Negelspach, Alisa Huskey, Kathryn Kennedy, Jungwon Cha, Jason Katz, William D S Killgore

**Affiliations:** University of Arizona, College of Medicine, Department of Psychiatry, Tucson, AZ, Pima County, United States; University of Arizona, College of Medicine, Department of Psychiatry, Tucson, AZ, Pima County, United States; University of Arizona, College of Medicine, Department of Psychiatry, Tucson, AZ, Pima County, United States; University of Arizona, College of Medicine, Department of Psychiatry, Tucson, AZ, Pima County, United States; University of Arizona, College of Medicine, Department of Psychiatry, Tucson, AZ, Pima County, United States; University of Arizona, College of Medicine, Department of Psychiatry, Tucson, AZ, Pima County, United States

**Keywords:** functional brain neuroimaging, functional connectivity, sleep deprivation

## Abstract

Functional neuroimaging conducted at regular intervals throughout sleep deprivation reveals key thalamocortical connectivity changes that characterize the transition from a well-rested to a sleep-deprived state. Decreased thalamic connectivity is distributed across sensorimotor, visual, and limbic networks, including subcortical structures such as the parahippocampal gyrus and hippocampus. Associated changes in globally efficiency closely track group-level deficits in psychomotor vigilance, suggesting that thalamic-cortical interactions play a role in wake maintenance during sleep deprivation. These patterns of connectivity disruptions may reflect transient, sleep-like states arising from unstable wakefulness. Causal modeling indicates impaired self-inhibition within the thalamus is a dominant feature of sleep deprivation, which likely contributes to wake-state instability and attentional lapses.

**Statement of Significance**

This study provides novel insights into the impact of sleep deprivation on thalamocortical connectivity, highlighting the thalamus as a central hub for changes in functional network topology under conditions of prolonged wakefulness. Decreases in thalamocortical connectivity poses important considerations for understanding the consequence of sleep-deprived cognitive fatigue and draws parallels to potential transient sleep states. These findings contribute to our understanding of individual variability in vulnerability to sleep deprivation and underscore the thalamus as a critical target for interventions aimed at mitigating cognitive decline during sleep deprivation.

## Introduction

Sleep is essential for maintaining brain health and optimal cognitive performance, yet individuals in modern society are frequently sleeping less than the recommended 7–9 h per night [1]. Inadequate sleep duration (and thus, prolonged wakefulness) degrades cognitive performance and increases the risk of attention lapses [2], maladaptive decision-making [3, 4], and psychiatric disorders [5]. Neuroimaging studies have provided key insights into how sleep deprivation disrupts brain function, revealing widespread alterations across large-scale cortical networks. A common theme that emerges in brain-wide analyses of individuals subjected to sleep deprivation is the upregulation of activity driven by subcortical structures, such as the thalamus, which plays a critical role in regulating arousal and maintaining stable brain-wide communication. Understanding the contributions of thalamocortical interactions in the context of sleep deprivation provides greater insight into the neural mechanisms underlying related cognitive deficits.

The thalamus has an established role in regulating wakefulness and arousal. Thalamocortical circuits facilitate communication between ascending arousal systems and cortical structures, enabling modulation of cortical activation [6]. When well-rested, this arousal mechanisms helps maintain cognitive abilities requiring alertness and sustained attention [7, 8]. Given that attentional lapses are a common cognitive deficit following sleep deprivation (for a review, see [9]), thalamic involvement is a likely a contributing factor to this impairment.

The thalamus’ role in regulating arousal is further implicated as a compensatory mechanism that maintains alertness during sleep deprivation. A meta-analysis of functional MRI literature shows that hyperactivation of the thalamus is a common result following sleep deprivation [10]. If increased thalamic activation is truly compensatory, then differences should show performance related differences throughout sleep deprivation. Indeed, Chee and colleagues found that lapses in a selective visual attention task during sleep deprivation were associated with lower thalamic activation than non-lapses [11]. Further evidence of the compensatory effects of thalamic activation can be derived from trait-based differences in resilience to sleep loss [12]. In a follow-up study, Chee and colleagues showed that individuals who were resilient to sleep loss were able to maintain a higher degree of thalamic activation during the visual selection task [13]. These findings suggest that thalamic compensation underlies a primary mechanism differentiating individuals who are more resilient to sleep, and thus able to perform better on tasks requiring sustained arousal/attention. Given the thalamus’s ability to modulate cortical activation, it’s compensatory response to sleep deprivation may also affect hemodynamic responses in task-related areas as an attempt to maintain cognitive functioning [14].

Thalamic involvement in the ability to stay vigilant extends beyond task-based activation and applies to thalamocortical interactions as well. Previous research suggests that connectivity between the thalamus and cortex is significantly impaired after extended wakefulness [15], and this correlates with next-day daytime sleepiness [16]. Maintaining arousal may depend not only on thalamic activation but also on its coordination with cortical regions. Given that the thalamus serves as a key relay region for sensory and arousal-related signals, its ability to sustain cognitive arousal is only effective insofar as it successfully transmits these signals to cortical regions responsible for vigilance and conscious attention. When thalamocortical connectivity is compromised, thalamic hyperactivation may fail to translate into functional compensation, leading instead to inefficient or maladaptive arousal regulation.

While the thalamus’ ability to help maintain arousal and cognitive performance during sleep deprivation has been well established, several key questions remain about this neurobiological mechanism. First, it is unclear whether changes in thalamocortical connectivity are a dominant feature of sleep deprivation or a peripheral effect. Previous methodological approaches involved selecting the thalamus region a priori, rather than taking an unbiased exploratory approach. Second, functional changes in thalamocortical circuits typically follow a before-and-after comparison paradigm. This binary approach makes it challenging to determine whether these changes occur gradually over time or are threshold dependent. Third, the causal role of thalamocortical connectivity in cognitive fatigue remains unclear. Specifically, it is unknown whether the thalamus drives abnormal cortical connectivity or whether cortical dysregulation alters thalamic function. Understanding the causal mechanisms of these changes would be useful for future development of targeted therapies which mitigate the debilitating effects of sleep deprivation in situations where it is unavoidable. We addressed these questions by conducting resting-state functional MRI scans at 6-h intervals throughout 37 h of sleep deprivation. Analyses were designed to address each question individually to determine: the defining pattern of changes in sleep-deprived connectivity patterns, the corresponding progression of thalamocortical disengagement, and the causal mechanisms of the breakdown in thalamocortical circuits.

## Materials and Methods

### Recruitment and screening

Participants (*n* = 20; female = 9; age = 23.6; *SD* = 4.7 years) were recruited using flyers, social media advertisements, and postings on the University of Arizona’s active studies webpage. Interested participants completed an online screening to exclude for mental illnesses and confirm MRI eligibility. Additional requirements included they must be primarily English speaking and right-handed. Following screening, participants were brought into the lab and administered a battery of neurocognitive assessments, underwent drug testing, and had a physical exam. Participants were then given a smartphone device for at-home monitoring of sleep and wake cycle via sleep diaries, caffeine consumption, and visual analogue fatigue scale [17]. Psychomotor vigilance tests (PVTs) were administered to participants twice a day (9 am and 8 pm) to determine baseline reaction times. Participants that maintained abnormal sleep schedules or reaction times were excluded from the study. Eligibility for the in-lab portion required 80 per cent of all queries be completed. A power analysis was conducted to ensure the final number of participants was sufficient based on previous measures of effect size in connectivity analyses following a period of sleep deprivation [18]. Power analysis indicated that a sample size of *n* = 20 was sufficient to detect effects at a significance level of *α* = .05.

### In-lab protocol

The in-lab portion of the study was conducted at the University of Arizona’s Center for Sleep, Circadian, and Neurosciences Research. On day 1, participants arrived at 2:00 pm for drug testing, physical screenings, and vital sign assessments. To assess neuropsychological status, a standard battery of subjective report questionnaires was administered at 6:00 and 9:00 pm After completing these tasks, participants followed their nighttime routine and were outfitted with polysomnography (PSG) electrodes at their scheduled bedtime of 10:30 pm.

On day 2, the sleep deprivation protocol began at 7:30 am. Participants woke up, completed their morning routine, showered, and ate breakfast. A baseline functional MRI scan started at 8:30 am and was repeated every 6 h. Following the baseline MRI, participants completed the Repeatable Battery for the Assessment of Neuropsychological Status [19] at 9:30 am and the Death Implicit Association Test (D-IAT) at 11:45 am to assess lucidity and suicidality risk [20]. Lunch occurred at 11:00 am in between tasks. Additional neurocognitive assessments were then administered at noon and repeated every 6 h thereafter. The second MRI session occurred at 2:30 pm, followed by a subsequent iteration of the standard battery (6:00 pm) and dinner at 6:30 pm. The day concluded with a third MRI session at 8:30 pm and a period of free time.

Day 3 followed a similar schedule to day 2, with MRI scans conducted at 6-h intervals (2:30 am, 8:30 am, 2:30 pm, and 8:30 pm) and the standard battery repeated at regular intervals (12:00 am, 6:00 am, 12:00 pm, and 6:00 pm). Other tasks and mealtimes remained consistent with day 2. The day ended with PSG and recovery sleep at the scheduled bedtime of 10:30 pm.

Day 4 followed a similar schedule, except with no additional MRI sessions. Participants completed the final iterations of the standard battery, and cognitive tasks at the corresponding times. Before being discharged, they underwent a medical examination by a licensed physician, including an ECG and vital sign assessment, to ensure their health and safety prior to returning home. This study protocol was reviewed and approved by the University of Arizona Internal Review Board for the protection of human subjects in research.

### Psychomotor vigilance task

The PVT score used in analyses consisted of mean response times during a standard ten-minute PVT with a random inter-stimulus interval of 2–10 s. Response times less than 150 ms were considered as false starts. Responses had to be logged within 4999 ms to be included in reaction time calculations.

### MRI acquisition

Participants were escorted to the MRI wing of the Biosciences Research Laboratory (Tucson, AZ) where scans were acquired with a 3T Skyra scanner (Siemens, Erlangen, Germany) equipped with a 32-channel head coil. Scanning sequences began with localizer scout image, followed by a T1-weighted 3D MPRAGE sequence (TR = 2.1 s, TE = 2.33 ms, flip angle = 12°, n-slice = 176, Voxel = 1 mm^3^). Phase encoding direction was A → P. The images were acquired with a field of view (FoV) of 256 mm.

Functional images were acquired using a T2^*^-weighted echo-planar imaging sequence. The imaging parameters were as follows: TR = 2000 ms, TE = 36.0 ms, flip angle = 90°, slice thickness = 2.0 mm, and voxel size = 2.0 × 2.0 × 2.0 mm^3^. A total of 60 slices were acquired in an interleaved order, with a FoV of 240 mm. The phase encoding direction was anterior to posterior (A → P). The scan used GeneRalized Autocalibrating Partial Parallel Acquisition (GRAPPA) parallel imaging with an acceleration factor of 2. The functional images were acquired over 180 volumes with a total scan duration of ~6 min.

### Preprocessing and analysis

Preprocessing was performed in the SPM12 [21] functional connectivity software CONN [22] (RRID:SCR_009550 Version: 22.v2407) [23] using the default preprocessing pipeline [24] which includes realignment, slice-timing correction (STC), normalization to MNI space, spatial smoothing, and denoising. CONN was specifically chosen for its standardized and well-documented preprocessing framework, updated methodology for physiological and motion artifact correction (e.g., aCompCor for physiological noise) [25], and its integration with common anatomical preprocessing tools such as fMRIPrep, ensuring consistency across datasets. Additional details of preprocessing and analyses utilized in this manuscript can be found below.

Functional and anatomical data were preprocessed including realignment with correction of susceptibility distortion interactions, STC, outlier detection, direct segmentation and Montreal Neurological Institute MNI-space normalization, and smoothing. Functional data were realigned using the Statistical Parametric Mapping (SPM) realign and unwarp procedure [26], where all scans were coregistered to a reference image (first scan of the first session) using a least squares approach and a six parameter (rigid body) transformation [27], and resampled using b-spline interpolation to correct for motion and magnetic susceptibility interactions. Temporal misalignment between different slices of the functional data was corrected following SPM STC procedure [28, 29], using sinc temporal interpolation to resample each slice blood oxygenation level-dependent (BOLD) time series to a common mid-acquisition time. Potential outlier scans were identified using ART [30] as acquisitions with framewise displacement above 0.9 mm or global BOLD signal changes above 5 standard deviations [31, 32]. Regressor files of outlier scans meeting these criteria which is then included as control covariates in subsequent analyses. Next, a reference BOLD image was computed for each subject by averaging all scans excluding outliers. Functional and anatomical data were normalized into standard MNI space, segmented into gray matter, white matter, and cerebrospinal fluid (CSF) tissue classes and resampled to 2 mm isotropic voxels following a direct normalization procedure [31, 33] using SPM unified segmentation and normalization algorithm [34, 35] with the default tissue probability map template. Last, functional data were smoothed using spatial convolution with a Gaussian kernel of 8 mm full width half maximum. Global signal regression was not included in preprocessing due to the potential to introduce spurious negative correlations, even in datasets where such anti-correlations may not exist biologically [36]. This is particularly problematic for investigations of thalamocortical connectivity, where decreases in functional coupling can be artificially diminished or inverted by global signal regression. Instead, more targeted and validated motion correction and physiological noise removal methods were employed to preserve inherent correlational structure within the data (see below).

Motion and physiological artifacts were removed using the regression of potential confounding effects characterized by white matter time series (5 CompCor noise components), CSF time series (5 CompCor noise components), motion parameters and their first order derivatives (12 factors) [37], outlier scans (below 15 factors) [32], session effects and their first order derivatives (14 factors), and linear trends (2 factors) within each functional run. Subsequently, BOLD signal was bandpass filtered [38] between 0.008 and 0.09 Hz. CompCor [39, 40] noise components within white matter and CSF were estimated by computing the average BOLD signal as well as the largest principal components orthogonal to the BOLD average, motion parameters, and outlier scans within each subject’s eroded segmentation masks. From the number of noise terms included in this denoising strategy, the effective degrees of freedom of the BOLD signal after denoising were estimated across all subjects [31].

Multivariate pattern analyses (MVPAs) were performed to estimate the first 10 eigenpatterns characterizing the principal sources of heterogeneity in functional connectivity across subjects and conditions. From these eigenpatterns, 10 associated eigenpattern-score images were derived for each individual subject and condition characterizing their brain-wide functional connectome. Eigenpatterns and associated scores were computed separately for each individual seed voxel as the singular value decomposition (group-level SVD) of functional connectivity values between this seed voxel and the rest of the brain. Functional MVPA for connectome inferences has been shown to be an effective method of assessing heterogeneity in connectome states and has been statistically validated using Monte-Carlo simulations [41]. To maintain consistency with functional preprocessing metrics such as MNI space conversion, and principal component physiological denoising, MVPA was conducted in CONN using the already calculated connectomes. The adjacency matrix threshold was set to 0.15, in conjunction with the default false discovery rate correction at *p* < .05 for statistical significance.

Seed-based connectivity maps and ROI-to-ROI connectivity matrices were computed for each participant by calculating bivariate correlations between BOLD time series from seed and target regions. Correlation coefficients are Fisher-transformed and a weighted general linear model (weighted-GLM) was applied to control for confounds. For each individual voxel, a separate GLM was estimated, with first-level connectivity measures at this voxel as dependent variables (one independent sample per subject and one measurement experimental condition), and specified covariates as independent variables. Voxel-level hypotheses were evaluated using multivariate parametric statistics with random-effects across subjects and sample covariance estimation across multiple measurements. Inferences were performed at the level of individual clusters (groups of contiguous voxels). Individual-level connectivity matrices were then entered into a second-level GLM for group-level statistical inference, enabling testing of average connectivity [24]. Cluster-level inferences were based on parametric statistics from Gaussian Random Field theory [24, 42]. Results were thresholded using a combination of a cluster-forming *p* < .001 voxel-level threshold, and a familywise corrected p-FWE < 0.05 cluster-size threshold [43]. Two participants were omitted from analysis due to incomplete functional datasets.

### Dynamic causal modeling

Spectral dynamic causal modeling (DCM) was used to determine effective connectivity among brain regions showing altered thalamocortical connectivity. This method was chosen given its validation in resting-state fMRI [44], and ability to accurately model statistical relationships between functionally connected regions [45]. In practice, spectral DCM estimates parameters that best equate the cross-spectral density of BOLD time series, thereby reproducing lag-order correlations of all connections including zero-lag (most closely related to functional connectivity). This enables a more direct hypothesis test of bidirectional influence between regions with known physical connections, rather than statistical dependencies which can be confounded by hidden states [46]. Furthermore, the deterministic model of spectral DCM integrates the hemodynamic response function to each region’s neuronal activity (hidden state), which essentially means that each region’s predicted neural fluctuations are convolved with a region-specific hemodynamic response function (HRF). As such, controlling for neurovascular coupling makes spectral DCM a more appropriate method to infer neural activity based on cross-spectral density between regions, if truly based on a biophysically plausible model. Finally, it is important to note that comparisons between established methods such as granger causality and the core methodology of DCM reach similar conclusions in conclusions of effective connectivity [47, 48].

Analytical procedures were based on the guidelines outlined in the SPM12 manual [49] as well as recently published guidelines for DCM [50, 51]. Briefly, BOLD signal extraction was performed using 6-mm spheres defined in the right thalamus, right postcentral gyrus, right angular gyrus, and right frontal pole. Regions of interest were chosen based on the combination of results from functional connectivity, and previous research on thalamocortical diffusion tensor tractography [52]: Areas that showed significant changes in functional connectivity were cross referenced with diffusion weighted connectivity of the thalamus to ensure an anatomically plausible biophysical model. Regions that were chosen based on this approach included the postcentral gyrus, frontal pole, and a portion of the angular gyrus/lateral occipital region. Using these four regions (including the thalamus), several models were compared on the basis of anatomic and functional connectivity using Bayesian model selection. Based on these results, a final reduced model was specified as the group causal model. Next, a parametric empirical Bayesian approach was used to estimate averages of effective connectivity parameters across participants, followed by Bayesian model reduction and averaging (BMR/BMA) to identify the specific connections that best, and most parsimoniously, explain group-level connectivity parameters. Connections were modeled bilinearly, which allowed for a more direct representation of functional connectivity results. Effective connectivity parameters reported from BMA were thresholded using free model energy for effective connectivity parameter >95 per cent. For additional information on the implementation of the Bayesian framework applied in effective connectivity, see Friston et al., 2015 [53].

## Results

### Functional connectivity MVPA results

**Figure 1:**
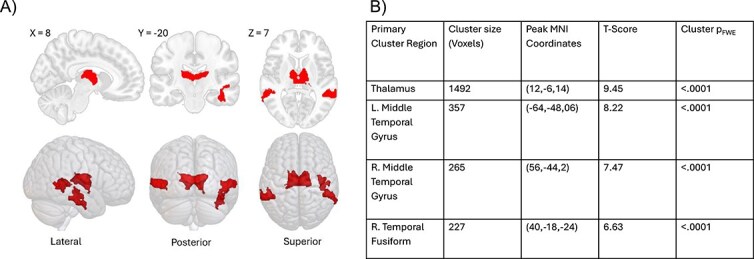
(A) Regions shown in red represent regions of interest in which patterns of connectivity explained the most variance across participants in the transition from well-rested to sleep deprived. (B) Table summarizes anatomical regions shown in the MVPA analysis. Peak MNI coordinates, and corresponding statistical values are reported for peak voxels.

We applied MVPA to identify regions capturing the dominant sources of heterogeneity in connectivity profiles between the well-rested baseline and the most sleep-deprived condition. The primary eigenvariate pattern revealed that prominent changes in bilateral thalamic connectivity accounted for the largest proportion of within-subject variance in the sleep-deprived state (Figure 1). Additionally, significant connectivity changes were observed in the mid-temporal lobes and right fusiform gyrus. These findings suggest that functional connectivity patterns involving these regions are among the most affected by the transition from well-rested to sleep-deprived states.

### Thalamic seed-to-voxel connectivity results

**Figure 2:**
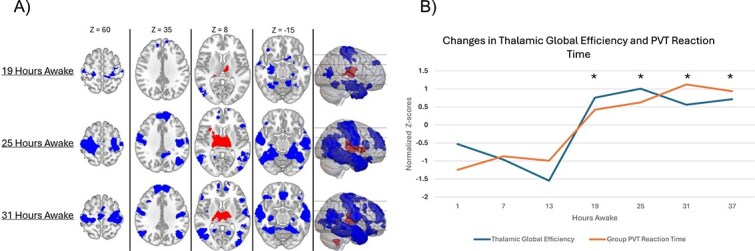
(A) Shows seed-to-voxel analysis of gradual changes in functional connectivity with the thalamus during sleep deprivation. Each timepoint is shown in comparison to baseline indicating which regions experienced a significant decrease in functional connectivity (shown in blue), and a significant increase in functional connectivity (shown in red). Individual voxel threshold is maintained at a *p* < .001. Each cluster shown meets significance after false discovery rate correction (*P*_fdr_ < .05). MNI coordinates of each axial slice is shown above each column. (B) ROI-ROI changes in thalamic global efficiency in and group PVT reaction time are shown at each timepoint. Group means are plotted over time and error bars reflect ±1 standard error of the mean (SEM). Data are plotted on a dual axis plot to show trends over the course of sleep deprivation. The *Y* axis on the left corresponds to reaction time in milliseconds, whereas the *Y* axis on the right corresponds to negative thalamic global efficiency. Asterisks indicate timepoints where thalamic global efficiency significantly deviated from baseline.

To investigate how thalamic connectivity patterns progressively changed during sleep deprivation, a seed-to-voxel analysis was performed using a single seed region derived from the averaged BOLD signal of both the left and right thalamus. Connectivity was compared at each 6-h interval relative to baseline. The results revealed widespread bilateral disruptions in the corticothalamic functional network (Figure 2A). Compared to well-rested baseline scans, several prominent bilateral regions showed a significant reduction in thalamocortical connectivity: the postcentral gyrus, precentral gyrus, angular gyrus, temporal poles, middle temporal gyrus, inferior frontal gyrus, and frontal poles. Additionally, subcortical regions such as the hippocampus and parahippocampal gyrus exhibited similar reductions in connectivity. Alongside these reductions, the thalamus demonstrated a marked increase in local connectivity, which persisted throughout the sleep deprivation period. Similar increases were also observed in the caudate and pallidum. A complete account of the anatomical results for the seed-to-voxel analyses are summarized in Supplementary Table S1.

The trend of thalamocortical connectivity changes over time were captured using a graph theory measure of global efficiency. Calculations for global efficiency included significant negative connectivity changes, capturing the overall trends in progressive thalamocortical disconnection. The maximum change in thalamocortical functional connectivity occurred at 25 h of extended wakefulness (*T* = 5.46, *p* < .05). To explore whether this change corresponded with behavioral performance, a post-hoc analysis was performed. Group changes in thalamocortical connectivity strongly correlated with deficits in mean reaction time on the PVT at each corresponding timepoint (*b* = 1444.6 ms, *R*^2^ = .74, *p* < .02). To further account for individual differences in thalamic connectivity, we conducted additional mixed-effects models examining the relationship between thalamic global efficiency and PVT measures across sessions. Increased negative thalamocortical network efficiency significantly predicted the slowing of psychomotor vigilance reaction time (*b* = 368.03 ms, *p* < .05) and deficits in sustained attention (*b* = 18.16 lapses, *p* < .05).

### Dynamic causal modeling

**Figure 3:**
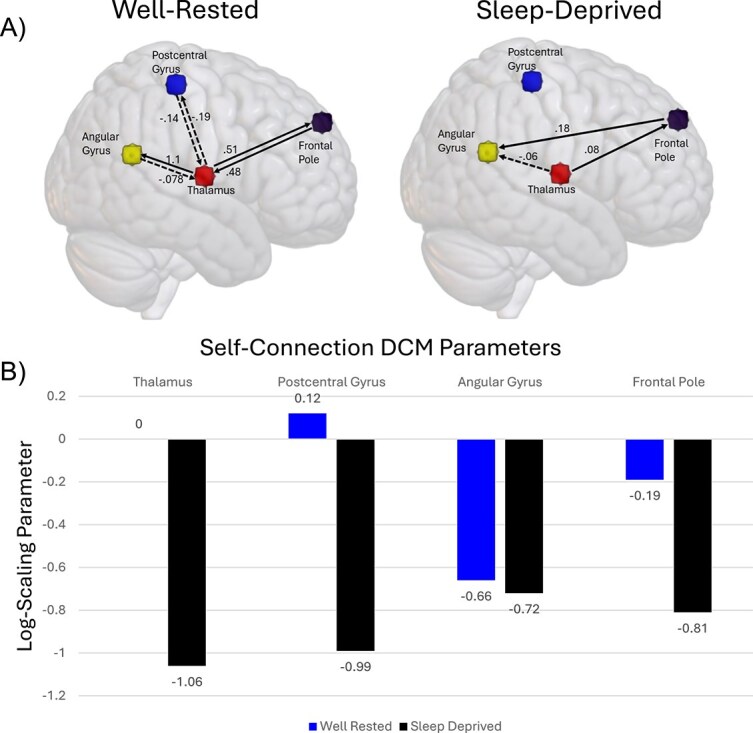
(A) Results from Bayesian model reduction and Bayesian model averaging of DCM in the well-rested and sleep-deprived conditions. Arrows represent directionality of effective connectivity parameters. ROI-ROI connections are expressed in units of Hz. Positive values indicate excitatory input (solid lines), whereas negative values (dashed lines) indicate inhibitory input. Timepoints used for effective connectivity analysis represent the most well-rested condition (1-h after wake) and most sleep-deprived condition (37-h after wake). (B) Bar graph shows the change in intrinsic connectivity parameter in the sleep-deprived condition relative to baseline. Units are expressed as log-scaling parameters of baseline-inhibitory connections. Each connection shown meets significance (free-energy >95 per cent).

Parametric empirical Bayesian estimation of group effective connectivity for the well-rested and sleep-deprived conditions also revealed dynamic changes in connectivity strength, as well as excitatory/inhibitory homeostasis (Figure 3). In the well-rested condition, the dominant thalamic connection was an excitatory input to the angular gyrus, with the angular gyrus in return having minimal inhibitory influence on the thalamus. In addition, the thalamus maintains bidirectional excitatory connections with the frontal pole, whereas bidirectional inhibitory connections exist between the postcentral gyrus and thalamus.

In the sleep-deprived condition, cortical input to the thalamus no longer meets significance (free-energy <95 per cent), indicating the presence of these connections no longer explained cross-spectral density changes in the spectral profile of thalamic BOLD signal. Furthermore, outgoing connections from the thalamus that remained significant were substantially altered: Thalamic input to the angular gyrus became negative, which suggests a shift to weak inhibition. Thalamic input to the frontal pole decreased from 0.51 to 0.08 Hz, indicating more lethargic responses. An excitatory connection also emerged in the sleep-deprived condition between the frontal pole and the angular gyrus. Finally, intrinsic connectivity in each region experienced a significant decrease in log-scaling parameters. This suggests that, in the sleep-deprived state, each ROI became less self-inhibitory relative to the implicit baseline. Notably, the thalamus experienced the most substantial reduction in the log-scaling factor, and thus a loss of self-inhibitory control.

## Discussion

Our results characterize dynamic changes in thalamocortical connectivity throughout sleep deprivation, providing critical insight into the neural mechanisms underlying cognitive impairments in attentional capacity. Functional connectivity MVPA provides strong evidence that altered thalamocortical connectivity is a defining feature differentiating sleep deprivation from a well-rested state. Seed-to-voxel analyses further specify the pattern of disruption, revealing that cortical regions abruptly disconnect from the thalamus in a manner consistent with changes in circadian arousal. The observed pattern of thalamocortical disruption is consistent with previous research of brain activity during wake–sleep transitions, indicating impaired thalamocortical connectivity may be the result of unstable wakefulness. Finally, DCM provided confirmatory evidence of thalamocortical disconnection and further indicates a loss of self-inhibitory control in both the thalamus and cortical areas. Ultimately, these results provide a clear characterization of the time-course, and pattern of thalamocortical impairments associated with psychomotor deficits following a period of sleep deprivation.

Functional MVPA has been employed to differentiate clinical phenotypes of individuals with sleep disorders from healthy controls. In conditions such as obstructive sleep apnea (OSA), where univariate analyses often fail to reveal clear group differences, MVPA has identified distributed patterns of altered connectivity that reliably distinguish individuals with OSA [54]. Of note, the thalamus and inferior temporal gyrus are included among the connections that differentiate OSA. Other studies investigating sleep deprivation specifically have also demonstrated the utility of MVPA for classifying individuals based on resilience to sleep loss. For instance, combined measures of white matter diffusivity within thalamocortical tracts have been used to classify vulnerability to sleep deprivation as measured by the PVT [55]. Our results remain consistent with previous research and provide additional insight into the changes in functional networks that occur as a result of sleep deprivation: MVPA results suggest that rather than a statistically significant effect, changes in thalamocortical connectivity are a dominant feature of sleep deprivation. In light of prior findings, this may reflect underlying reductions in wake stability and alertness. In addition to the thalamus, mid-temporal regions and the right fusiform gyrus also show altered connectivity patterns across participants, suggesting that these regions may also contribute to the transition into a sleep-deprived state. Future research should explore how connectivity changes in these areas relate to corresponding deficits in cognitive functioning, beyond attention, due to sleep deprivation.

Seed-to-voxel analyses further inform the MVPA, by identifying specific cortical regions that become progressively disconnected from the thalamus due to sleep deprivation. Initially, the most disrupted thalamocortical connections are found within somatosensory, visual, and limbic networks, suggesting that early-stage impairments primarily affect sensory processing and emotional regulation. With prolonged wakefulness, connectivity reductions extend to lateral occipital, parietal, and frontal regions, indicating a breakdown in sensorimotor integration and decision-making. These findings are consistent with previous sleep deprivation research linking cognitive impairments to sensorimotor integration and adaptive decision-making [3, 4, 56]. Interestingly, the later involvement of frontal regions in thalamic seed-to-voxel analyses suggests that executive functions may be initially spared, potentially as a compensatory mechanism to sustain cognitive performance in more resilient individuals. This hypothesis is supported by prior research showing that sleep deprivation increases thalamic influence on the frontoparietal network, which predicts inter-individual differences in PVT lapses [57].

Significant reductions in thalamic connectivity were also observed with the bilateral mid-temporal lobes, parahippocampal gyrus, and hippocampus—regions crucial for memory encoding, spatial navigation, and contextual processing. These findings align with prior research indicating that sleep deprivation weakens hippocampal and parahippocampal activation, contributing to episodic memory deficits [58]: Thalamic disengagement from these memory-related structures may underlie the weakened activation, thereby contributing to working memory impairments observed in sleep-deprived individuals.

In examining the progression of thalamocortical connectivity throughout sleep deprivation, we observed that functional connections remained stable until ~19 h of wakefulness. From thereon out, several cortical areas exhibited a significant reduction in their interaction with the thalamus which persisted for the remainder of the experimental protocol. The abrupt disconnection aligns with previous interpretations of a compensatory mechanism to counteract the breakdown of wakefulness. The timing of this threshold dependent effect provides further insight of cognitive processes involved. The most significant decrease in thalamocortical connectivity occurred abruptly after the wake maintenance zone, where rising homeostatic sleep pressure is offset by increased circadian arousal and sustained attention performance [59, 60]. The fact that such an abrupt change in thalamocortical connectivity occurred subsequent to the wake maintenance zone suggests that circadian arousal involves thalamocortical circuitry. Supporting this, a study by Muto and colleagues demonstrated circadian rhythmicity in thalamic activation closely mirroring the melatonin rhythm [61]. Individual differences in circadian rhythmicity may contribute to variance in the presented connectivity analyses and are acknowledged as a limitation. Future studies should investigate whether diurnal changes in thalamic connectivity are stably associated with objective measures of circadian phase.

The widespread alterations in thalamocortical connectivity may have far-reaching consequences on cognition, extending beyond simple deficits in reaction time. Considering that the thalamus plays a critical role in gating transitions between wakefulness and sleep-like states [62–64], it is possible that altered thalamic connectivity is conducive, or perhaps enables, transient microsleeps during wakefulness. Comparing these results with EEG/fMRI recordings of sleep–wake transitions offer a means to test this hypothesis. First, large-scale resting-state analyses suggest that functional connectivity data captures unstable wakefulness, including transient states resembling early-stage sleep [65]. Notably, these transitions are marked by reduced subcortical–cortical connectivity, particularly in the frontal, parietal, and temporal cortices, aligning closely with regions showing significantly decreased thalamic-cortical connectivity. Second, an fMRI/EEG study on the transition from wakefulness to sleep indicates that this process is characterized by reduced thalamocortical connectivity and increased intra-thalamic coherence [66]. This result closely aligns with the functional connectivity findings of this study: The thalamus displays increased local connectivity following sleep deprivation. Given these parallels, these changes may be characteristic of a cognitive state with unstable wakefulness. These changes in thalamic network connectivity are plausibly conducive for aberrations in a stable wake state. However, without simultaneous EEG recordings there is no way to corroborate specific moments of potential microsleep episodes. Other factors, such as sensory gating, or compensatory network reorganization are alternative plausible interpretations of these changes in patterns of functional connectivity.

DCM provides an additional dimension of how thalamocortical communication is impaired using a state-space model of cross-spectral densities [67]. In the well-rested state, the thalamus maintained significant bidirectional feedback with the postcentral gyrus, angular gyrus, and frontal pole. However, under sleep deprivation, cortical input no longer exerted a significant influence on the spectral profile of thalamic BOLD signal. This result further corroborates the observed decreased functional connectivity while also indicating a loss of cortical input to the thalamus. Additionally, a novel effective connection emerged in the sleep-deprived condition: a significant excitatory input from the frontal pole to the angular gyrus. Altered effective connectivity between the angular gyrus and frontal cortex mirrors findings from insomnia research: In insomnia disorder, the angular gyrus exhibits hyperconnectivity with several regions including the frontal cortex [68]. The aforementioned pattern of altered connectivity also correlated with insomnia severity and hyperarousal. In a recent clinical trial, inhibition of the angular gyrus (inferior parietal lobule) with transcranial magnetic stimulation improved polysomnographic measures in a sample with insomnia symptoms [69]. Together, this evidence points to the prefrontal/angular gyrus as undergoing changes in network organization as well as effective connectivity in response to sleep loss. Theoretically, if experienced chronically, these changes could become potentiated, serving as a precipitating factor in the etiology of insomnia.

An intriguing observation from DCM was the marked reduction in intrinsic self-inhibition within both the thalamus and associated cortical regions during sleep deprivation. This disrupted self-regulation was most pronounced in the thalamus, potentially contributing to weakened thalamocortical functional connectivity. However, the chronological order of the breakdown in connectivity is not entirely clear. In other words, whether this loss of thalamic self-inhibition leads to reduced cortical input, or the loss of cortical input results in reduced thalamic regulation remains a question of interest. Intracellular recordings of the thalamus suggest that reductions in cortical drive induce hyperpolarization, leading to self-sustained delta oscillations—a shift from tonic-wake-like firing to rhythmic burst activity [70]. Compared to tonic-wake-like activity, this slower rhythmic activity profile would theoretically reduce the effective decay rate, which is consistent with the shift in the log-scaling factor of intrinsic connectivity within the thalamus. Thus, the loss of thalamic self-inhibition can reasonably be interpreted as a transition to a more sleep-prone state, where sustained rhythmic activity may persist without strong self-inhibition.

Disruptions in thalamocortical connectivity following sleep deprivation reflect a breakdown in thalamic arousal mechanisms of the cortex and impaired psychomotor vigilance. Regions showing decreased thalamic connectivity are, unsurprisingly, involved in a range of neurocognitive abilities shown to be affected by sleep deprivation [71]. The pattern of functional connectivity that become altered due to sleep deprivation bear striking similarities to neuroimaging markers of unstable wakefulness and wake-to sleep transitions. Extrapolating from this, impaired thalamocortical connectivity may increase the likelihood of transient sleep-like activation, thereby impairing one’s ability to maintain cognitive functions requiring sustained vigilant attention. Further investigation is required to validate this interpretation. The transition from a well-rested to a sleep-deprived state is also accompanied with significant changes in effective connectivity. The thalamus in particular experienced a prominent decrease in log-scaling factor indicating a decrease in self-inhibition. In summary, the transition from well-rested to sleep deprived is characterized by an abrupt and progressive breakdown of thalamocortical connectivity, which correlates with slowed stimulus responses, attentional lapses, and is accompanied by impaired self-inhibition.

### Limitations

The primary informative signal assessed by functional MRI is change in BOLD, which is an indirect measure of neuronal activity, and is inferred through neurovascular coupling [72–74]. Thus, results stemming from functional analyses of connectivity throughout sleep deprivation are an indirect measure of neural processing between the thalamus and cortical regions, which primarily reflects changes in blood oxygenation. As such, potential confounds in neurovascular coupling, which may occur during sleep deprivation, may contribute to error variance within the data. It should be noted that functional connectivity analyses are inherently robust to global influences on BOLD signal amplitude, and preprocessing procedures were included to control for physiological noise (e.g., aCompCor). Nonetheless, diurnal variations in hemodynamic responses cannot be entirely eliminated. Thus, we acknowledge that factors such as changes in blood pressure, and other physiological rhythms, may contribute to BOLD fluctuations within the data. In addition, the temporal resolution (TR = 2) in this dataset may exceed the entirety of transient cognitive lapses such as microsleep states. As such, cognitive lapses may be incompletely represented in BOLD signal changes within each acquisition. Future studies of thalamocortical contributions to arousal deficits during sleep deprivation would benefit from simultaneous EEG/fMRI recordings. Finally, a consideration of individual circadian modulation and neurovascular coupling could further improve the validity of functional analyses.

## Supplementary Material

Supplementary_Table_1_zpaf065

## Data Availability

Data presented in this manuscript will be made available upon reasonable request to the corresponding author, provided that the requested data does not contain any identifying personal health information, in accordance with HIPAA and related participant protection policies.
