# Supplementary material for: Breakdown of thalamocortical connectivity under sleep deprivation: implications for cognitive arousal and transient sleep states
Source: Sleep Adv. 2025 Oct 1;6(4):zpaf065. doi: 10.1093/sleepadvances/zpaf065 (PMC12551454; doi:10.1093/sleepadvances/zpaf065)
Supplement: Supplementary_Table_1_zpaf065 [file supplementary_table_1_zpaf065.pdf]

Breakdown of Thalamocortical Connectivity Under Sleep Deprivation: Implications for Cognitive Arousal and Transient Sleep States

David Negelspach[a], Alisa Huskey[a], Kathryn Kennedy[a], Jungwon Cha[a], Jason Katz[a], William D.S. Killgore[a]

a: University of Arizona, College of Medicine, Department of Psychiatry, Tucson, Arizona, Pima County, USA

Corresponding Author: David Negelspach, Ph.D., davidnegelspach@arizona.edu

Anatomical Region	MNI Coordinates (x, y, z)	Number of Voxels	First Appearance (Session)
Postcentral Gyrus (L)	(-38, -26, +54)	1552	Session 5
Postcentral Gyrus (R)	(+42, -22, +52)	1508	Session 5
Precentral Gyrus (R)	(+38, -16, +56)	1467	Session 6
Precentral Gyrus (L)	(-32, -16, +56)	1409	Session 6
Temporal Pole (R)	(+44, +14, -32)	1061	Session 4
Middle Temporal Gyrus, Posterior (L)	(-60, -26, -10)	990	Session 6
Temporal Pole (L)	(-44, +14, -32)	909	Session 4
Lateral Occipital Cortex, Superior (L)	(-48, -68, +26)	787	Session 5
Frontal Pole (L)	(-12, +54, +34)	695	Session 6
Thalamus (L)	(-8, -16, +10)	695	Session 5
Middle Temporal Gyrus, Posterior (R)	(+58, -18, -12)	637	Session 6
Thalamus (R)	(+10, -14, +10)	563	Session 5
Temporal Occipital Fusiform Cortex (R)	(+34, -50, -18)	553	Session 5
Lateral Occipital Cortex, Inferior (R)	(+52, -68, +4)	512	Session 4
Angular Gyrus (R)	(+54, -54, +20)	494	Session 6
Frontal Pole (R)	(+14, +50, +30)	490	Session 6
Lateral Occipital Cortex, Superior (R)	(+50, -66, +22)	432	Session 5
Angular Gyrus (L)	(-48, -56, +22)	419	Session 6
Temporal Occipital Fusiform Cortex (L)	(-34, -54, -16)	415	Session 6
Middle Temporal Gyrus, Anterior (R)	(+58, -2, -24)	395	Session 6

Middle Temporal Gyrus, Anterior (L)	(-58, -4, -20)	362	Session 6
Hippocampus (R)	(+26, -18, -18)	352	Session 4
Superior Frontal Gyrus (L)	(-6, +42, +44)	347	Session 6
Temporal Fusiform Cortex, Posterior (R)	(+36, -30, -24)	345	Session 5
Parahippocampal Gyrus, Anterior (R)	(+24, -12, -30)	267	Session 4
Lingual Gyrus (R)	(+18, -50, -8)	254	Session 5
Hippocampus (L)	(-26, -22, -18)	247	Session 6
Temporal Fusiform Cortex, Posterior (L)	(-36, -36, -22)	237	Session 5
Cerebellum 4/5 (L)	(-12, -50, -14)	227	Session 6
Supplementary Motor Cortex (L)	(-2, -4, +62)	199	Session 6
Lateral Occipital Cortex, Inferior (L)	(-46, -78, +8)	196	Session 6
Lingual Gyrus (L)	(-16, -50, -10)	191	Session 5
Superior Frontal Gyrus (R)	(+8, +40, +48)	182	Session 6
Parahippocampal Gyrus, Posterior (L)	(-24, -32, -18)	174	Session 6
Middle Temporal Gyrus, Temporooccipital (R)	(+54, -56, +8)	167	Session 6
Superior Temporal Gyrus, Posterior (L)	(-60, -24, -2)	152	Session 6

Supplementary Figure 1. Brain regions showing impaired functional connectivity with the bilateral thalamus relative to baseline. For each cluster, anatomical location and peak MNI coordinates are reported. Statistical maps were thresholded at the voxel level at $p < 0.001$, with clusters surviving family-wise error correction at $p < 0.05$. Only clusters larger than 50 voxels are shown. The session comparison at which each significant change appeared is indicated.
